# Deletion of the MBII-85 snoRNA Gene Cluster in Mice Results in Postnatal Growth Retardation

**DOI:** 10.1371/journal.pgen.0030235

**Published:** 2007-12-28

**Authors:** Boris V Skryabin, Leonid V Gubar, Birte Seeger, Jana Pfeiffer, Sergej Handel, Thomas Robeck, Elena Karpova, Timofey S Rozhdestvensky, Jürgen Brosius

**Affiliations:** Institute of Experimental Pathology (ZMBE), University of Münster, Münster, Germany; University of Cambridge, United Kingdom

## Abstract

Prader-Willi syndrome (PWS [MIM 176270]) is a neurogenetic disorder characterized by decreased fetal activity, muscular hypotonia, failure to thrive, short stature, obesity, mental retardation, and hypogonadotropic hypogonadism. It is caused by the loss of function of one or more imprinted, paternally expressed genes on the proximal long arm of chromosome 15. Several potential PWS mouse models involving the orthologous region on chromosome 7C exist. Based on the analysis of deletions in the mouse and gene expression in PWS patients with chromosomal translocations, a critical region (*PWScr*) for neonatal lethality, failure to thrive, and growth retardation was narrowed to the locus containing a cluster of neuronally expressed *MBII-85* small nucleolar RNA (snoRNA) genes. Here, we report the deletion of *PWScr*. Mice carrying the maternally inherited allele (*PWScr^m−/p+^*) are indistinguishable from wild-type littermates. All those with the paternally inherited allele (*PWScr^m+/p−^*) consistently display postnatal growth retardation, with about 15% postnatal lethality in C57BL/6, but not FVB/N crosses. This is the first example in a multicellular organism of genetic deletion of a C/D box snoRNA gene resulting in a pronounced phenotype.

## Author Summary

Prader-Willi syndrome, or PWS, is a complex neurogenetic disorder and the most common genetic cause of life-threatening childhood obesity. Newborns have poor muscle tone, making suckling difficult, which leads to poor weight gain. After infancy, they experience extreme hunger, leading to obesity. Other symptoms include short stature, mental retardation, and often infertility. In PWS patients, a complex set of genes on the paternal chromosome 15 (in the PWS region) is missing or unexpressed. In an attempt to understand this disorder, various protein-coding genes in this region have been deleted in mice, but none of the resulting phenotypes consistently correlated with the human disease. This region also contains a cluster of genes that encode functional non-protein-coding RNAs. We deleted specifically the MBII-85 small nucleolar RNA (snoRNA) gene cluster on the parental mouse chromosome, which did not affect expression of any of the other snoRNA or protein-coding genes in the PWS region. These mice consistently displayed postnatal growth retardation starting from day 5 to 6, low postnatal lethality only in certain genetic backgrounds (<15%), and no adolescent obesity. Thus, this mouse model, with the deletion of a small, brain-specific non-protein-coding RNA, should prove useful for teasing out the various molecular pathologies of PWS.

## Introduction

**Figure 1:**
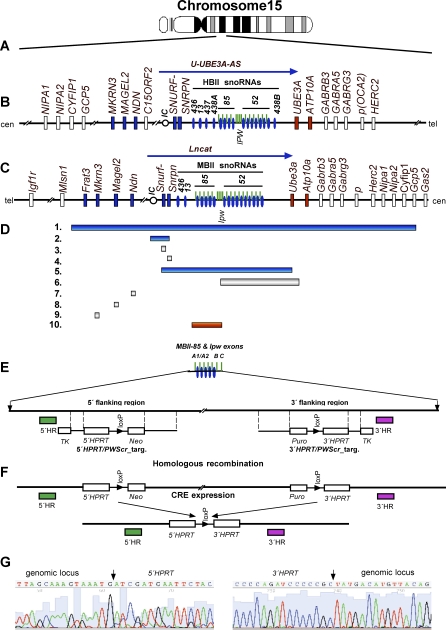
Genome Organization of the PWS/AS Locus in Man and Mouse (A) Structure of human chromosome 15. (B) Schematic representation of the human 15q11-q13 region (not drawn to scale). The centromeric (cen) and telomeric (tel) positions are labeled. PWS protein coding genes and snoRNA genes are marked as boxes and ovals, respectively. Paternally, maternally, and biparentally expressed genes are labeled blue, red, and white, respectively. *IPW* exons of the paternally expressed, imprinted U-UBE3A-AS transcript are indicated by green bars and a blue arrow, respectively. The imprinting center (IC) is indicated with a small circle. The snoRNA genes: *HBII-436*, *HBII-13*, *HBII-437, HBII-438A, HBII-85. HBII-52* and *HBII-438B* are labeled as *436*, *13*, *437, 438A, 85, 52* and *438B*, accordingly. (C) Corresponding mouse syntenic chromosome 7C region. The paternally expressed Lncat transcript is indicated by a blue arrow. (D) Deletions in the PWS/AS region. (1) Insertion of the Epstein–Barr virus Latent Membrane Protein 2A, (*LMP2A*) transgene into the PWS/AS locus resulted in deletion of approximately 4 megabases between the *Mlsn1* and *Gas2* genes [12,29]. Depending on the parental origin it causes PWS or AS symptoms in mice. (2) The 42 kb *Snrpn* promoter PWS-IC deletion resulted in loss of imprinting and a PWS-like phenotype in mice [13]; analogous deletion of the PWS-IC and *Snrpn* gene up to exon 7 results in a paternal to maternal imprint switch and leads to PWS [40]. (3) Paternal *Snrpn* exon 2 deletion [14] produced no abnormal phenotype. (4) Mice with a deletion of 1kb (exons 5–7) of the *Snrpn* gene are phenotypically normal [13]. (5) Paternal deletion from *Snrpn* to *Ube3a* resulted in a PWS-like phenotype in mice [14]. (6) Deletion affecting npc *Ipw* exons and *MBII-52* snoRNA gene cluster after paternal inheritance produced no PWS-like phenotype [15]. (7) Targeting of the *Necdin* gene produced either a PWS-like phenotype [21,23] or no obvious phenotype [22]. The discrepancies were explained by different strategies in *Necdin* targeting and different mouse genetic backgrounds [15]. (8) Inactivation of the *Magel2* gene produced altered behavioral rhythmicity [19]. (9) Deletion of the *Mkrn3* (*Zfp127*) gene had no apparent phenotypic effect [15]. (10) PWS critical region (*PWScr*). It has been proposed that deletion of this region should play a major role in the development of PWS [16,24]. Paternal deletion of *PWScr* causes growth retardation in mice (this study). (E) Locus containing the cluster of *MBII-85* snoRNA genes and *Ipw* exons, and its deletion targeting strategy using “chromosome engineering” [26]. Filled boxes represent 5′HR and 3′HR DNA probes (labeled green and purple respectively). Targeting constructs *5′HPRT/PWScr*_targ and *3′HPRT/PWScr*_targ are indicated. Open boxes correspond to the Thymidine kinase gene (*TK*), Neomycin resistance gene (*Neo*), Puromycin resistance gene (*Puro*), as well as the 5′ and 3′ parts of the *Hypoxanthine Phosphoribosyl Transferase* gene (*HPRT*). LoxP sites are indicated by arrows. (F) Structure of the *PWScr* after two consecutive HR events, and CRE recombinase-induced deletion. (G) Sequencing of integration sites after HR and deletion of the *PWScr* confirmed the anticipated deletion at the nucleotide level. Extended sequences (Genbank accession number EU233428) of regions flanking the *HPRT* cassette insertion are presented in Figure S1.

The human genetic locus 15q11-q13 is subject to genomic imprinting that is controlled by a bipartite imprinting center (IC) [1]. Imprinting defects or chromosomal rearrangements/deletions within this locus (Figure 1) are responsible for the development of two clinical disorders—Angelman (AS) and Prader-Willi (PWS) syndromes. AS (MIM 105830) is a complex neurogenetic disorder characterized by mental retardation, severe limitations in speech and language and abnormal behavior, and results from loss of maternal expression of the *Ube3A* gene [2]. PWS (MIM 176270) is a complex neurogenetic disorder with a population prevalence of 1 in 10 000 to 50 000 [3–5] that is characterized by decreased fetal activity, muscular hypotonia, failure to thrive, short stature, obesity, mental retardation, and hypogonadotropic hypogonadism, for review, see [2,6]. PWS results from lack of paternal expression of one or several imprinted genes within the PWS/AS locus. Several paternally expressed protein-coding genes map to this locus, including *NECDIN (NDN)*, *MAGEL2*, *MKRN3*, and the bi-cistronic *SNURF-SNRPN* (Figure 1B). There are also numerous paternally expressed C/D box snoRNA genes located downstream from the *SNURF-SNRPN* gene. Most of them are organized into two main clusters of *HBII-85* and *HBII-52* snoRNAs, containing 29 and 47 copies, respectively. Other snoRNAs are present as single (*HBII-436*, *HBII-13* and *HBII-437*) or double copy (*HBII-438a*/*438b*) genes (Figure 1B). Most, if not all, snoRNAs are processed from a long, non-protein-coding RNA (npcRNA) transcript designated U-*UBE3A-ATS* in human and *Lncat* (large paternal non-protein-coding RNA, encompassing *Snurf-Snrpn* and *Ipw* exons together with the *Ube3a* antisense transcript) in mouse [7–9]. U-*UBE3A-ATS* extends ∼450 kb from the untranslated U exons upstream of the small nuclear ribonucleoprotein N (*SNURF/SNRPN*) gene to the *UBE3A* gene (Figure 1B).

In mouse the syntenic PWS/AS locus is located on chromosome 7C and contains all the aforementioned protein coding and non-protein-coding gene orthologs, except for the presence of protein-coding gene *Frat 3* and absence of *HBII-437* and *HBII-438a*/*438b* snoRNA genes (Figure 1C). Several mouse models for PWS have already been generated (Figure 1D). They can be divided into 3 groups: 1) transgenic mouse lines with disruptions of the PWS/AS locus; 2) mice with targeted elimination of the imprinting center (IC) controlling transcription of PWS genes, or targeted elimination of individual, single genes from the PWS locus; and 3) mice with uniparental paternal disomy (UPD) [10,11].

In all existing PWS mouse models (Figure 1D) that involve large deletions comprising several paternally expressed, imprinted genes, severe phenotypes (failure to thrive and early postnatal lethality) were observed [10,12–15]. Targeting of the single *Mkrn3* and *Snrpn* genes, or some of the *Ipw* exons together with the *MBII-52* snoRNA genes cluster [13,15–17] or deletion of an analogous region in human [18] all produced no PWS-like phenotypes. Elimination of the *Magel* 2 gene caused altered behavioral rhythmicity (Figure 1D8) [19]. However this gene is unlikely to be a main “PWS player” [20]. Currently, only elimination of the single *Necdin* gene leads to the development of early postnatal lethality and neurological abnormalities resembling the PWS, although the phenotypic effect depends on the targeted region and genetic background of the mice, some of which had no apparent phenotype [21–23]. The PWS critical region (*PWScr*) was narrowed to the locus containing the *MBII-85* small nucleolar RNA (snoRNA) gene cluster based on existing mouse models [16] and gene expression analysis in PWS patients with chromosomal translocations [18,24,25]. These studies indicate that *SNURF/SNRPN*, *MKRN3*, *NECDIN* and *MAGEL2* genes are unlikely to play a primary role in the pathogenesis of PWS. However, the question of their possible functional contribution to more severe phenotypic expression seen in typical PWS patients remains open [16,18,24]. We have applied the “chromosome engineering” technique [26] to delete the *PWScr* in mice. When the deleted allele is inherited maternally (*PWScr^m−/p+^*), no phenotypic abnormalities are visible. When it is inherited paternally (*PWScr^m+/p−^*), we consistently observe postnatal growth retardation in mice and less than 15 percent postnatal lethality in 129SV x C57BL/6 genetic crosses.

## Results/Discussion

**Figure 2:**
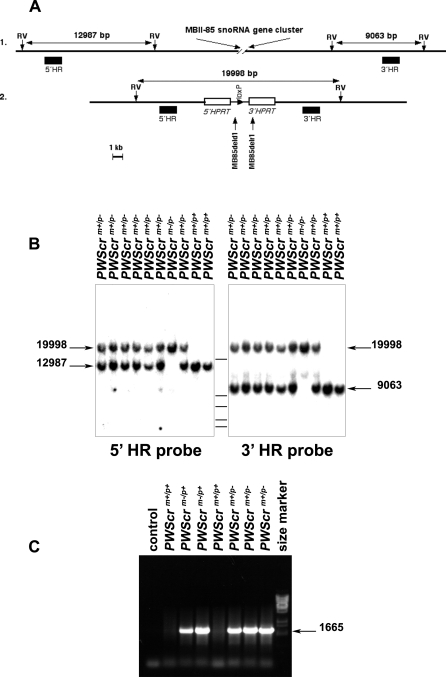
Analysis of Mice with the *PWScr* Deletion (A) Schematic representation of 1. wild-type and 2. *PWScr* targeted alleles. DNA fragments are labeled according to Figure 1E and 1F. *Eco*RV (RV) restriction endonuclease sites are designated by vertical arrows. Sizes of diagnostic restriction fragments are indicated. The positions of PCR primers MB85deld1 and MB85delr1 are shown. (B) Southern blot analysis. *Eco*RV digested DNA samples of individual mice (genotype indicated on top) were hybridized with 5′HR and 3′HR probes. Bands representing the wild-type alleles (12987 bp for 5′HR and 9063 bp for 3′HR) and the *PWScr*-deleted allele (19998 bp) are indicated by arrows. Size markers (14140, 8453, 7242, 6369, and 5687 bp) are represented by horizontal bars between the two blots. (C) Genotyping by PCR analysis using primers MB85deld1/MB85delr1 (positions are shown in A). Deletion of *PWScr* results in a PCR product of 1665 bp. Genotypes of mice are indicated.

To test the hypothesis that the *PWScr* is the most probable candidate region for neonatal lethality, failure to thrive and postnatal growth retardation, we devised the following strategy for producing *PWScr^m+/p−^* mice (Figure 1E and 1F). Hypoxanthine-guanine phosphoribosyltransferase (*HPRT*)-deficient ES cells, AB2.2, were modified through homologous recombination (HR) using targeting constructs 5′*HPRT*/*PWScr*_targ and 3′*HPRT*/*PWScr*_targ to place loxP sites proximal to the 5′ flanking region of the *MBII-85* snoRNA gene cluster and distal to the *Ipw* exon C, respectively (Figure 1E). Deletion of the *PWScr* harboring the entire cluster of *MBII-85* snoRNA genes together with *Ipw* exons A-C was accomplished by expressing CRE recombinase in ES-targeted cells (Figures 1F and 2A) and injecting these into blastocysts. We identified two chimaeras derived from one of the *PWScr*-deleted ES clones with successful germ-line transmission. Deletion of the *PWScr* allele was confirmed several ways: 1) Resistance of ES cells to HAT media requires a functional *HPRT* gene. Restoring the intact *HPRT* gene through deletion of the *PWScr* leads to resistance of ES cells to HAT media. 2) Southern blot analysis of all HAT-resistant ES colonies (data not shown), as well as all *PWScr*-deleted mice, using 5′HR and 3′HR probes (Figure 2A and 2B), revealed identical bands corresponding to the correctly deleted *PWScr* allele (19998 bp). 3) We PCR amplified and sequenced flanking regions of the *PWScr* locus together with the inserted *HPRT* gene (Figures 1G, 2C, and S1) using PCR primers MB85seqD1 and MB85seqR1 (Table S1), and confirmed the deleted *PWScr* allele as well.

**Figure 3:**
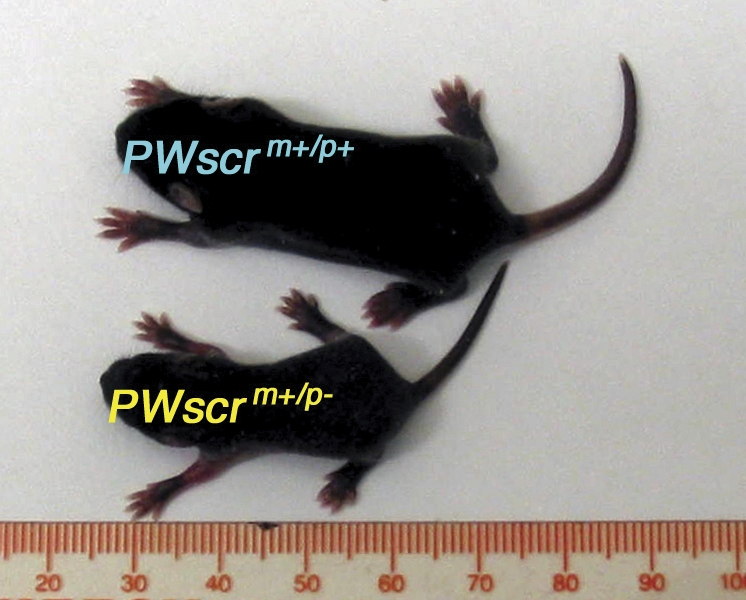
Growth Differences among *PWScr^m+/p−^* and *PWScr^m+/p+^* Siblings Representative pair of mice from the same litter at postnatal day 10 (129SV x C57BL/6 genetic crosses).

**Figure 4:**
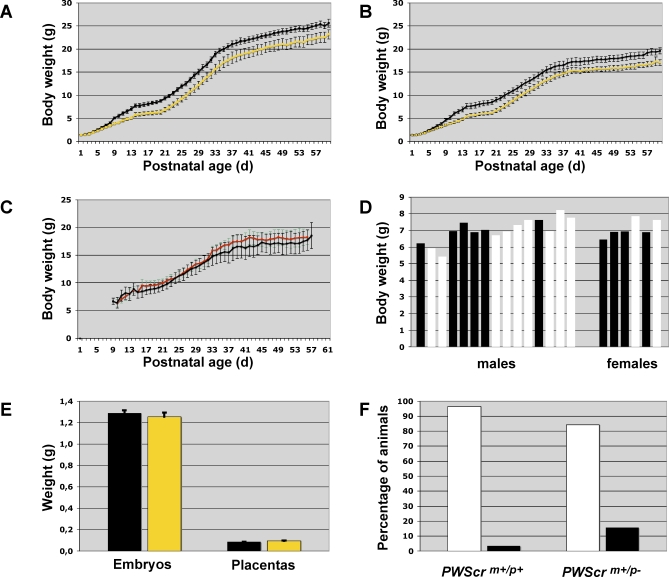
Growth Retardation and Postnatal Lethality in *PWScr^m+/p−^* Mice (129SV x C57BL/6 Genetic Background) (A) Growth dynamics of 82 investigated male mice. The yellow line corresponds to weight gain of 34 mice with the *PWScr*-deleted allele; black bars are statistically significant intervals (confidence level 95%, p=0.05). The black line corresponds to 48 wild-type males; black bars are statistically significant intervals (p=0.05). (B) Growth dynamics of 82 investigated female mice. The yellow line corresponds to weight gain in 38 females with the *PWScr*-deleted allele; black bars are statistically significant intervals (p=0.05). The black line corresponds to 44 wild-type females; black bars are statistically significant intervals (p=0.05). (C) Growth dynamics of 40 female mice with the maternally transmitted *PWScr*-deleted allele. The red line corresponds to weight gain in 16 females with the *PWScr*-deleted allele; green bars are statistically significant intervals (p=0.05). The black line corresponds to 24 wild-type females. (D) Growth comparison of 15-day-old control mice with wild-type mice. Mice, containing the *5′HPRT/PWScr*_targ cassette in the *PWScr* locus (*5′HPRT^m−/p+^*; 6.898±0.257 g), are represented by white bars, and wild-type mice (6.711±0.129 g) by black bars (p=0.2751). (E) Embryo weights (E18.5) and the corresponding placenta weights (n=15 for all groups). The weights of embryos and placentas from *PWScr^m+/p+^* and *PWScr^m+/p−^* mice are indicated with black and yellow bars, respectively. Embryo weights E18.5 *PWScr^m+/p+^* 1.290±0.105 g (mean±SD), *PWScr^m+/p−^* 1.253±0.151 g, (p=0,934); placenta weights E18.5 *PWScr^m+/p+^* 0.086±0.012 g, *PWScr^m+/p−^* 0.094±0.021 g, (p=0.290). (F) Postnatal lethality of *PWScr^m+/p−^* (n=64) and wild type *PWScr^m+/p+^* (n=57) mice in 129SV x C57BL/6 (>85% C57BL/6 contribution) genetic crossings (p<0.02). White and black bars represent percentages of surviving and dead mice, respectively, after 90 days.

*PWScr^m+/p−^* pups born from chimaeras were significantly smaller than their wild-type siblings, such that on postnatal day 10, the *PWScr^m+/p−^* individuals (Figure 3) could be reliably predicted prior to DNA analysis. We monitored the weights of all mice over several weeks after continuous breeding and found that only the *PWScr^m+/p−^* and *PWScr^m−/p−^* mice displayed postnatal growth retardation compared to the *PWScr^m+/p+^* siblings. Statistical analyses were performed on mice separated by genotype, genetic background and gender (Figures 4A–4D and S2), as well as separately for litters (data provided upon request). Postnatal growth retardation in *PWScr^m+/p−^* mice was observed, thus far, over six generations, independent of genetic background [e.g., 129SV x C57BL/6 (>85% C57BL/6 genetic background contribution, Figure 4A and 4B), 129SV x C57BL/6 x FVB/N (∼50% FVB/N contribution, Figure S2A and S2B), and 129SV x C57BL/6 x BALB/c (∼50% BALB/c contribution, Figure S2C and S2D)]. Differences in growth dynamics between *PWScr^m+/p+^* and *PWScr^m−/p−^* or *PWScr^m+/p−^* mice continued to be statistically significant into adulthood (up to 1 year in BALB/c crosses) (Figure 4A, 4B, and S2). Moreover, not a single case of obesity was detected. Interestingly, when growth dynamics for mice were analyzed by gender, deficiencies in the *PWScr^m+/p−^* or *PWScr^m−/p−^* female mice tended to be less pronounced than those in the male deletion mice (Figure 4A, 4B, S2C, and S2D). This observation is well correlated with a statistical analysis of PWS patients indicating that the degree of short stature is more prominent in males than in females, irrespective of ethnic groups (genetic background) [27]. Furthermore, mice with the maternally transmitted *PWScr*-deleted allele were indistinguishable from their wild type littermates (Figure 4C). In addition, males (*5′HPRT*) derived from 5′*HPRT*/*PWScr*_targ-targeted ES cells were crossed with C57BL/6 females. As expected, we did not observe growth retardation in the *5′HPRT^m−/p+^* mice (p=0.275) (Figure 4D).

Notably, the observed postnatal growth retardation phenotype became apparent during the first week of life starting from postnatal day 5 in males and 6 in females (Figure 4A and 4B; Table S2), while there were no growth differences at early postnatal ages P1-P4 (Figure 4A and 4B; Table S2). In agreement with the absence of early postnatal growth retardation, no weight differences were observed during embryonal development at E12.5, E15.5 and E18.5, or in late gestation (E15.5 and E18.5) placentas (Figure 4E, and data not shown). Hence, one possible explanation for our observations might be poor breast-feeding in *PWScr^m+/p−^* pups as is the case with postnatal PWS patients. Insufficient milk intake resulting in growth retardation is consistent with the Holland hypothesis and observations in TgPWS mice (transgenic deletion PWS mouse model; Figure 1D1) suggesting, that the main basis of the PWS syndrome is not obesity and uncontrollable craving but early postnatal starvation [28,29].

The *PWScr^m+/p−^* mice exhibit postnatal growth retardation, but contrary to early predictions [16] postnatal lethality was observed in only 15 percent of the cases in 129SV x C57BL/6 genetic crosses (Figure 4F). On the other hand, we did not observe postnatal lethality in the FVB/N crosses. *PWScr^m+/p−^* mice were dying from postnatal days 1 to 22. The surviving *PWScr^m+/p−^* and *PWScr^m−/p−^* mice are alive and apparently well for at least 1 year. In future experiments, it would be interesting to test the possible role of MBII-13 and MBII-436 snoRNAs and/or the genomic region between the *Snrpn* and *MBII-85* genes for their contributions to more severe phenotypes with higher rates of postnatal lethality. In addition, our *PWScr^m+/p−^* mouse model is in good agreement with two recently identified patients with a balanced chromosomal translocation involving *SNRPN* [24,30]. Both patients lack *HBII-85* expression and exhibit a mild PWS phenotype, (e.g., they did not require gavage feeding, but were growth retarded.)

PWS is also characterized by hypogonadotropic hypogonadism and infertility in patients [6]. We studied the fertility of the *PWScr^m+/p−^* and *PWScr^m−/p+^* mice and observed that *PWScr^m+/p−^* males and females transmitted the *PWScr* deleted allele to offspring. To further extend our observations, we established 10 breeding pairs from each of the *PWScr^m+/p−^* and *PWScr^m−/p+^* males with 80 wild-type females. All matings resulted in pregnancies leading to successful live births with litter sizes around eight (7.93 ± 0.46 for *PWScr^m+/p−^* and 8.33 ± 0.48 for *PWScr^m−/p+^* males), and always included wild-type mice and mice carrying the *PWScr*-deleted allele in a ratio of 1:1 (4.17 ± 0.34 : 3.66 ± 0.38 for *PWScr^m+/p−^* and 4.00 ± 0.54 : 4.17 ± 0.53 for *PWScr^m−/p+^*; Table S3). Thus, male mice containing the *PWScr*-deleted allele inherited maternally or paternally, are transmitting this allele in a Mendelian fashion.

**Figure 5:**
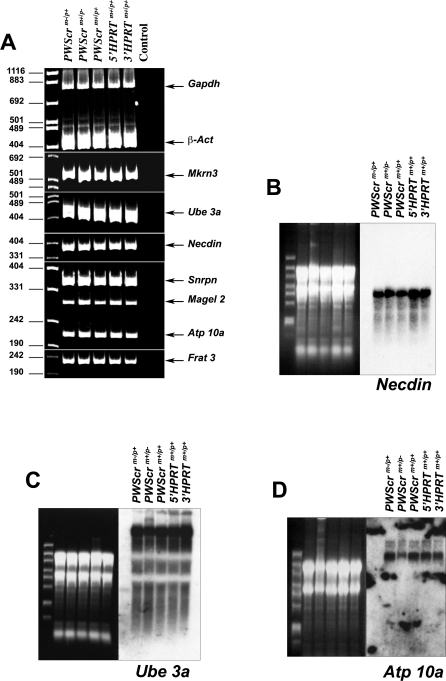
mRNA Analysis of PWS and AS Imprinted Genes in *PWScr*-Deleted Mice (A) RT-PCR analysis. Mouse genotypes are indicated. *5′HPRT^+/+^* and *3′HPRT^+/+^* correspond to homozygous *5′HPRT/PWScr*_targ or *3′HPRT/PWScr*_targ targeted mice, respectively; size markers are given in bp at the left; the position of RT-PCR products corresponding to individual transcripts are marked by arrows. Gapdh and β-Act transcripts were used as controls. (B–D) Northern blot analyses of selected mRNAs from the PWS locus. Ethidium bromide-stained RNA gels (prior to blotting) are shown as RNA loading controls. RNA size markers are as follows: 6000, 4000, 3000, 2000, 1500, 1000, 500, and 200 nt.

The PWS locus includes several paternally expressed, protein coding genes, including *Necdin*, *Magel2*, *Mkrn3*, *Frat3*, and the bi-cistronic *Snurf-Snrpn*. To examine whether the deletion of the paternal *PWScr* from mouse chromosome 7C perturbed the expression of the aforementioned imprinted genes, we analyzed their expression levels in our mice by RT-PCR and real-time PCR. We failed to detect any significant differences in the expression levels of the investigated genes in *PWScr^m+/p−^* mice compared to control littermates *PWScr^m+/p+^* (Figure 5A; Table 1). In addition, the controversial data concerning the involvement of *Necdin* in the PWS phenotype [21–23] prompted us to also examine its expression in more detail by Northern blot hybridization (Figure 5B). Consistent with the RT-PCR and real-time PCR results, there were no differences in the levels of Necdin mRNA in brains of the *PWScr^m+/p−^*, *PWScr^m−/p+^* or control *PWScr^m+/p+^*, *5′HPRT^m+/p+^* and *3′HPRT^m+/p+^* mice (Figure 5B; Table 1 and data not shown).

**Table 1:**
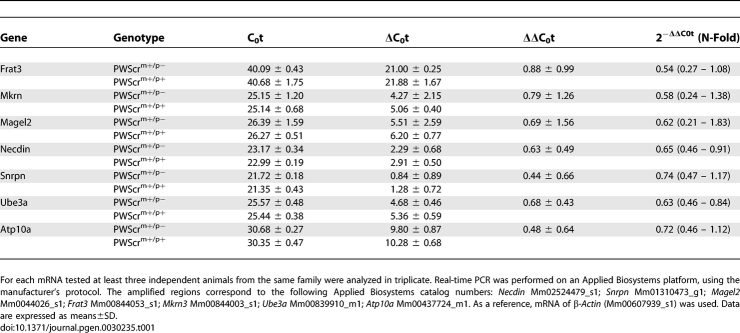
C_0_t, ΔC_0_t, ΔΔC_0_t and N-Fold Values for mRNA Levels from the PWS/AS Locus Obtained with Real-Time PCR

We also examined the levels of the maternally expressed, imprinted *Ube3a* and *Atp10a* protein coding genes, located at the end of the PWS locus and involved in the development of AS [2] (Figure 1B and 1C). The RT-PCR (Figure 5A), real-time PCR (Table 1), and Northern blot hybridization (Figure 5C and 5D) analyses revealed similar expression levels of both genes in brains of *PWScr^m+/p−^* and *PWScr^m−/p+^*, as well as in control mice.

**Figure 6:**
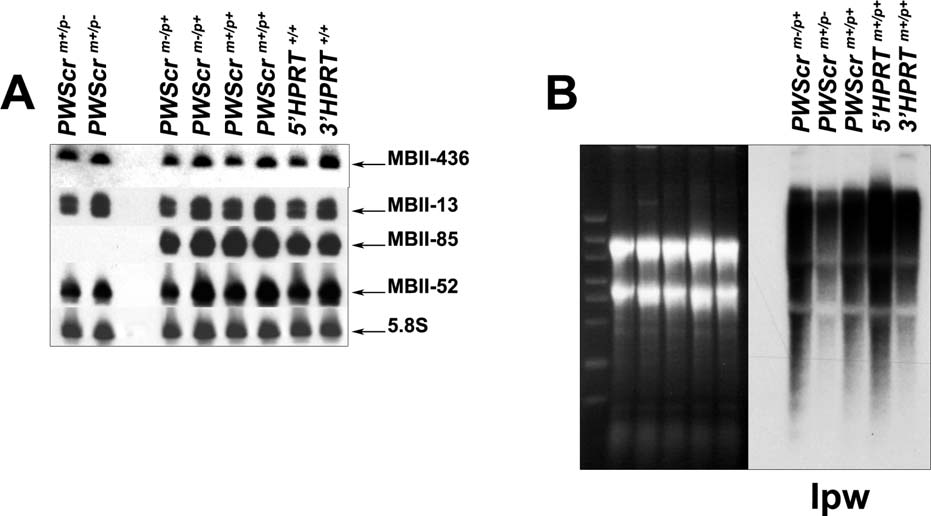
Expression of snoRNAs and Ipw Transcripts in *PWScr*-Deleted Mice (A) Expression analysis of snoRNAs from the PWS locus. 5.8S rRNA was probed as RNA loading control. Mouse genotypes are indicated (see Figure 5). (B) Northern blot analysis of Ipw transcripts. Ethidium bromide-stained RNA gels (prior to blotting) are shown as RNA loading controls. RNA size markers are identical to those in Figure 5.

The mouse PWS locus on chromosome 7C also contains numerous neuronal, paternally expressed C/D box snoRNA genes, including two single copies of *MBII-436* and *MBII-13*, and two multiple copy clusters, *MBII-85* and *MBII-52* [7,31]. Vertebrate snoRNAs are embedded in introns of protein coding genes, which are posttranscriptionally processed to yield mature mRNA and snoRNA. Occasionally the spliced exons are devoid of open reading frames, as if the sole function of the transcript is the expression of the snoRNA [32]. This is apparently the case for most, if not all, snoRNAs from the PWS locus that are co-transcribed with the large paternally expressed polycistronic Lncat npcRNA [7–9]. The Lncat transcript is complex and generates, by alternative splicing and other processing events, numerous RNA products (e.g., those represented by expressed sequenced tags (ESTs) and mature snoRNAs). The corresponding *Ipw* exons are a subset of Lncat*-*derived ESTs and map to the MBII-85 and MBII-52 snoRNA clusters that are interspersed with repeated exons A1/A2 and G1/G2, respectively [31,33]. The *Ipw* exons B, C, H, E, F map between both clusters. Northern blot analyses of RNA samples extracted from brains of *PWScr^m+/p−^* mice revealed the complete absence of MBII-85 snoRNA while expression of all other snoRNA genes in the PWS locus were unaffected (Figure 6A).

**Figure 7:**
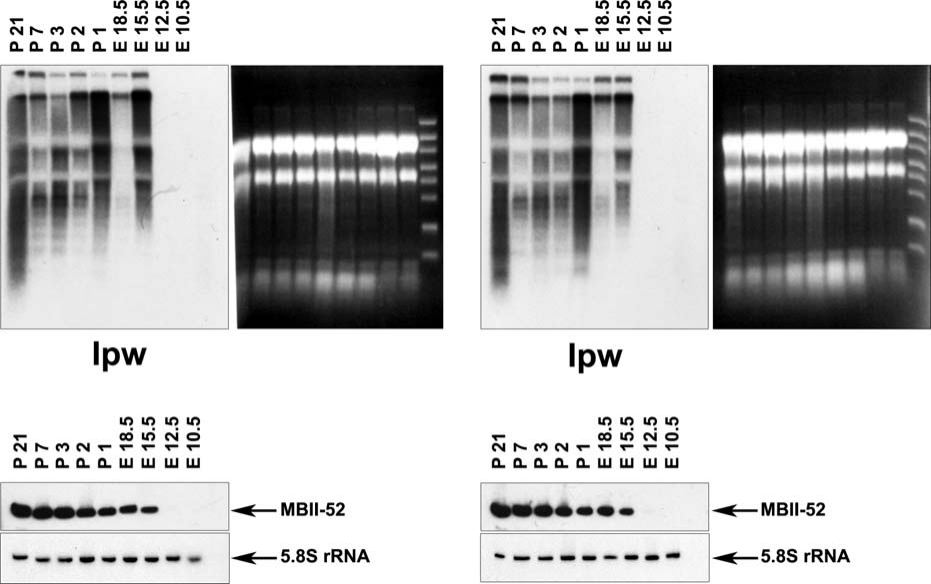
Expression of Ipw Exons and MBII-52 snoRNA throughout Different Stages of Wild-Type Mouse Development (Two Independent Experiments Using RNAs from WT Mice) We examined the expression patterns of Ipw exons (*Lncat* derived ESTs) and MBII-52 snoRNAs by Northern blot hybridization using total RNA from embryonal (E) and postnatal (P) brain, except for embryonic days 10.5 and 12.5, when total RNA was isolated from whole embryos. As loading control 5.8S rRNA was probed. Signals appeared between E12.5 and E15.5 days (note that this observation may, in part, be due to the fact that we switched from whole embryos to brains as sources of total RNA). Interestingly, while expression of MBII-52 snoRNA constantly increased, the levels of transcripts harboring Ipw exons fluctuated. This might indicate that maturation of MBII-52 snoRNA is not dependent solely on Ipw exon processing and that snoRNAs might be derived from Lncat npcRNA via alternative pathways.

We then analyzed the expression of the *Ipw* exons using Northern blot hybridization with a cDNA probe containing the F and G exons, and found it to be slightly decreased in *PWScr^m+/p−^* mice (Figure 6B). However, expression of MBII-52 snoRNA was not altered, presumably because the primary Lncat transcript can undergo different processing pathways to yield mature MBII-52 snoRNA (Figure 7). Recently it was reported that lack of *HBII-52* snoRNA genes together with most of the *IPW* exons did not result in a PWS phenotype in either human individuals [18] or in a mouse model [15,16]. Therefore, it is less likely that deletion of alternatively spliced *Ipw* exons A1/A2, B and C are responsible for the phenotype we obtained here, although we cannot completely exclude the possibility that lack of those exons in a long npcRNA can contribute to it. In future experiments, we will address the question, whether expression of MBII-85 snoRNA in a different host gene is sufficient to compensate the observed phenotype.

Lack of MBII-85 snoRNA expression in *PWScr^m+/p−^* compared to *PWScr^m−/p+^* mice, along with the unaltered expression of paternally and maternally expressed genes from the PWS/AS locus in both heterozygous mice, indicates that deletion of *PWScr* does not affect imprinting of neighboring genes. Moreover, patients with translocations leading to a similar deletion encompassing the genes encoding HBII-85 and HBII-438A snoRNA exhibit normal biparental methylation patterns [24,30]. Thus, absence of *MBII-85* snoRNA is the most likely cause for the phenotype observed in *PWScr^m+/p−^* and *PWScr^m−/p−^* mice (Figure 6A).

In Eukarya, most C/D box npcRNAs guide site-specific 2′-O-methylation in rRNAs and small nuclear RNAs (snRNAs) by complementarity to defined sites within these RNA targets [34]. However, most if not all C/D box small npcRNAs that map to the PWS locus, including MBII-85 and MBII-52 snoRNAs, lack significant complementarities to any rRNA or snRNA targets [7,31]. Although a major role for MBII-52 in the etiology of the PWS can be excluded, cell culture experiments have suggested that MBII-52 snoRNA might play a role in A to I editing and/or alternative splicing of the 5HT-2c serotonin receptor pre-mRNA [35,36]. In spite of these discrepancies, one might still be tempted to propose that MBII-85 snoRNA interacts (by complementarity) with a yet unidentified RNA target. However, other less orthodox functions for MBII-85 snoRNA and its role in postnatal growth retardation must also be seriously entertained. In any event, *MBII-85* is the first example of a C/D box snoRNA gene, whose deletion results in an obvious phenotypic change in a multicellular organism. Future experiments might reveal additional, less obvious defects and deficiencies in *PWScr^m+/p−^* mice. Our mouse model will serve as an important tool for further investigations of the molecular pathogenesis of PWS in man.

## Materials and Methods

### Targeting vectors.

For construction of the 5′*HPRT*/*PWScr_*targ and 3′*HPRT*/*PWScr_*targ targeting vectors, we isolated clones RPCIP711K19517Q6 and RPCIP711J18414Q6, respectively, from the RPCI21 mouse PAC library (RZPD German Resource Center for Genome Investigation) using the MBII-85 oligonucleotide (Table S1). The 5′*HPRT*/*PWScr_*targ construct was generated using the 5′ flanking region of the *MBII-85* gene cluster as a template for PCR-amplification of 1075 bp and 7387 bp DNA fragments with primer pairs 5′FLAdir/5′FLArev and 5′FLBdir/5′FLBrev, respectively. The PCR fragments were used as homologous arms in the targeting vector. The cassette containing the 5′ portion of the *HPRT* gene, the loxP site, and the neomycin resistance gene was subcloned from a λ phage vector kindly provided by A. Bradley (Baylor College of Medicine, Houston, USA). The *thymidine kinase* (*TK*) gene was placed outside of the homologous arm (Figure 1E).

The 3′*HPRT*/*PWScr_*targ construct was cloned in a similar way. The 3′ flanking region of the *MBII-85* gene cluster was used as a template to PCR-amplify 1098 bp and 6720 bp DNA fragments using primer pairs 3′FLBdirN/3′FLBrev and 3′FLAdir/3′FLArev, respectively. The insertion cassette, containing the 3′ portion of the *HPRT* gene, the loxP site, and the *puromycin resistance* gene was subcloned from the λ phage vector kindly provided by A. Bradley. The *TK* gene was placed outside of the homologous arm.

### Cloning of 5′HR and 3′HR probes for DNA blot hybridization.

The 5′ HR probe for Southern blot hybridization was generated by using the PAC DNA containing the 5′ region of the *MBII-85* gene cluster in two PCR reactions with oligonucleotide pairs 5′PRAdir/5′PRArev and 5′PRBdir/5′PRBrev, and ligating and cloning the resulting PCR products into the pSL300 vector [37]. The 3′ HR probe was cloned in a similar fashion. PCR products were obtained with oligonucleotide pairs 3′PRAdir/3′PRArev and 3′PRBdir/3′PRBrev (Table S1).

### ES cell transfection, selection of targeted clones and blastocyst injections.

*HPRT*-deficient embryonic stem cells AB2.2 (from A. Bradley), passage 17, were expanded in HEPES-buffered, Dulbecco's modified Eagle's medium supplemented with 15% fetal bovine serum (HyClone), nonessential amino acids, L-glutamine, β-mercaptoethanol, 1000 U/ml recombinant LIF (Chemicon) and antibiotics (penicillin 100 U/ml and streptomycin 100 μg/ml) on a γ-irradiated monolayer of SNL6.7 cells (from A. Bradley) or mouse primary fibroblasts. For electroporation, 2 × 10^7^ ES cells were resuspended in 20 mM HEPES pH 7.4, 173 mM NaCl, 5 mM KCl, 0.7 mM Na_2_HPO_4_, 6 mM dextrose, and 0.1 mM β-mercaptoethanol [38]. The *Not*I linearized replacement targeting vectors 5′*HPRT*/*PWScr*_targ, 3′*HPRT*/*PWScr*_targ, and intact CRE expressing cassette pOG231 (55 μg DNA of each) were electroporated at 25 μF and 400V (Gene Pulser; Bio-Rad). After electroporation, cells were plated onto 100 mm culture dishes containing a γ-irradiated monolayer of primary, G-418-resistant, or SNL6.7 (G-418, puromycin- and HAT-resistant) fibroblast feeder cells. Thirty-eight hours later, 350 μg/ml G418 (Invitrogen) and 0.2 μM 2′-deoxy-2′-fluoro-β-D-arabinofuranosyl-5-iodouracil (FIAU) (Moravek Biochemicals and Radiochemicals, USA) or 1.0 - 0.5 μg/ml puromycin (Sigma) and 0.2 μM FIAU, or HAT (Sigma) were added to the culture medium. The medium was replaced every day and colonies were picked and analyzed eight days after plating. *5′HPRT loxP*-targeted ES cells were analyzed using oligonucleotide pairs 85–5′screen2d/85–5′screen2r and 85–5′screen3d/85–5′screen3r for a nested PCR approach. For analysis of *3′HPRT loxP*-targeted cells we employed the 85–3′screen1d/85–3′screen1r and 85–3′screen2d/85–3′screen2r nested PCR primer pair combinations. DNA blot analysis was performed as described [39]. Membranes were hybridized with ^32^p−labeled 5′HR and 3′HR probes (Figure 2A). Several independent ES clones containing the CRE-mediated *PWSc*r-deletion were injected into 3.5-day-old B6D2F1 (C57BL/6 x DBA) blastocysts, and the resulting embryos were transferred to CD-1 foster mice. Chimeras were identified by their agouti coat color.

### PCR amplification and sequencing flanking regions of PWScr.

The 5.7 kb DNA fragment containing flanking regions of *PWScr* together with the inserted *HPRT* gene was amplified and sequenced using PCR primers MB85seqD1 and MB85seqR1. Sequencing reactions were performed using the BigDye terminator cycle sequencing reaction kit (PE Applied Biosystems) and resolved on an ABI Prism 3100 (Perkin Elmer) capillary sequencing machine.

### PWScr mice genotyping.

To genotype mice we performed PCR analysis of genomic DNA from tail biopsies using the primer pair MB85deld1/MB85delr1 (Figure 2A and 2C; Table S1). PCR cycling was done at 93 °C – 2 min; 7 cycles (93 °C – 40 sec, 70^ o^C – 20 sec, touch down −1 °C, extension 67^ o^C – 1 min 40 sec); 35 cycles (93 °C – 40 sec, 55^ o^C – 20 sec, 67^ o^C – 1 min 40 sec). The final extension was performed at 67 °C for 5 min.

### RT PCR and Northern blot analysis.

Total RNA was isolated from mouse brains using TRIzol reagent (Invitrogen) according to the manufacturer's instructions. RNA samples, 20 μg each, were treated with RNase-free DNase I (Roche). First strand cDNA synthesis was performed using Transcriptor reverse transcriptase (Roche) and hexamer oligonucleotides, followed by PCR amplification with gene specific oligonucleotides (Table S1). The cDNA probes for necdin, Ube3A, Atp10a mRNAs, and Ipw exons were PCR amplified, cloned in the pCRII vector (Invitrogen), and subsequently sequenced using gene specific oligonucleotides (Table S1). Approximately 20 μg of total RNA was denatured, fractionated on 1.2% agarose formaldehyde gels, and transferred to GeneScreen nylon membranes (NEN DuPont). Hybridization was performed with ^32^P-labeled cDNA probes. Northern blot analysis of snoRNAs was performed with specific oligonucleotides (Table S1) as described [31].

### Mice.

A *PWScr*-deficient mouse line was established by breeding male chimeras nos. 2 and 5 from one mutant ES cell line with female C57BL/6 mice to produce heterozygous mice. Subsequently, heterozygous mice were interbred or bred to C57BL/6 mice. All breeding occurred at the ZMBE animal facility of the University Clinics, Münster in a controlled (21 °C, 30–50% humidity) room with a 12:12 hour light-dark cycle, and mice were housed under non-enriched, standard conditions in individually ventilated (36 (l) x 20 (w) x 20 (h) cm) cages for up to five littermates. Pups were weaned 19 – 23 days after birth and females were kept separately from males.

### Statistical analysis.

Statistical analysis was performed using the StatView software package. Body weight was analyzed using Student's *t*-test, or ANOVA for each day of postnatal age. Weights of placentas and embryos were analyzed using Mann-Whitney nonparametric statistics. Postnatal lethality was analyzed with the chi-square test.

## Supporting Information

Figure S1 Sequences at the Site Replacing the *PWScr* with the *HPRT* Cassette The primary sequence of the 5′- (chr7:67,076,064) and 3′- (chr7:66,883,161) regions flanking the inserted *HPRT* cassette (Genbank accession number EU233428). The sequence of *Ipw* exon H is underlined. The sequence positions correspond to the UCSC Genome Browser (mouse, July 2007 assembly). The compiled deleted region is estimated to span ∼193 kb; however, because the gap size is only estimated (∼50 kb chr7:66,952,205–67,002,206), the region deleted in our mouse model could span from 143 to ≥193 kb.
(82 KB PDF)

Figure S2 Growth Retardation in *PWScr^m+/p−^* Mice in FVB/N and BALB/c Genetic Crosses (A, B) Growth dynamics of mice in 126SV x C57BL/6 x FVB/N genetic crosses (∼50% FVB/N contribution) beginning at postnatal day 1.
(A) Growth dynamics of 11 investigated males. The yellow line corresponds to the weight gain of 8 *PWScr^m+/p−^* males. The black line corresponds to the weight gain of 3 wild-type males.
(B) Growth dynamics of 21 investigated female mice. The yellow line corresponds to the weight gain of 13 *PWScr^m+/p−^* females. The black line corresponds to the weight gain of 8 wild-type females.
(C, D) Growth dynamics of mice in 126SV x C57BL/6 x BALB/c genetic crosses (∼50% BALB/c contribution) beginning at postnatal week 6.
(C) Growth dynamics of 100 analyzed male mice. The yellow line corresponds to weight gain of 47 *PWScr^m+/p−^* males. The black line corresponds to 53 *PWScr^m+/p+^* males.
(D) Growth dynamics of 102 female mice. The yellow line corresponds to the weight gain of 51 *PWScr^m+/p−^* females. The black line corresponds to the weight gain of 51 *PWScr^m+/p+^* females. In all cases, black error bars exhibit statistically significant intervals (confidence level 95%, p=0.05).
(109 KB PDF)

Table S1 Sequences of Oligonucleotides Used in This Study (27 KB DOC)

Table S2 Statistical Analysis (ANOVA) of Early Postnatal Weight Gain for *PWScr^m+/p+^* and *PWScr^m+/p−^* Mice Separated by Gender P1 – P8 indicate the corresponding postnatal days. N is the number of mice investigated from each genotype. Mean is the average mouse weight per category in grams. Statistically significant differences (p < 0.05) are indicated in bold.
(76 KB DOC)

Table S3 Number of Embryos/Pups from Several *PWScr^m+/p−^* and *PWScr^m−/p+^* Male Mice Twenty-nine crosses between *PWScr^m+/p−^* males and wild type BALB/c females resulted in 230 embryos/pups. Among those 106 were identified as *PWScr^m+/p+^*, 121 as *PWScr^m+/p−^* and 3 were not genotyped. We have performed 12 crosses between *PWScr^m−/p+^* males and wild type BALB/c females and obtained 100 embryos/pups. Fifty were identified as *PWScr^m+/p+^*, 48 as *PWScr^m+/p−^* and 2 were not genotyped.
(63 KB DOC)

## Abbreviations

AS: Angelman syndrome
FIAU: 2′-Deoxy-2′-fluoro-β-D-arabinofuranosyl-5-iodouracil
IC: imprinting center
Lncat: large paternal non-protein-coding RNA (encompassing *Snurf-Snrpn* and Ipw exons together with the Ube3a antisense transcript)
npcRNA: non-protein-coding RNA
PWS: Prader-Willi syndrome
*PWScr*: Prader-Willi syndrome critical region
TgPWS: transgenic mouse with insertion of the LMP2A (Epstein–Barr virus latent membrane protein 2A) transgene into the PWS/AS locus
UPD: uniparental paternal disomy
