# Supplementary material for: Deletion of the MBII-85 snoRNA Gene Cluster in Mice Results in Postnatal Growth Retardation
Source: PLoS Genet. 2007 Dec 28;3(12):e235. doi: 10.1371/journal.pgen.0030235 (PMC2323313; doi:10.1371/journal.pgen.0030235)
Supplement: Figure S1 — The primary sequence of the 5′- (chr7:67,076,064) and 3′- (chr7:66,883,161) regions flanking the inserted HPRT cassette (Genbank accession number EU233428). The sequence of Ipw exon H is underlined. The sequence positions correspond to the UCSC Genome Browser (mouse, July 2007 assembly). The compiled deleted region is estimated to span ∼193 kb; however, because the gap size is only estimated (∼50 kb chr7:66,952,205–67,002,206), the region deleted in our mouse model could span from 143 to ≥193 kb. (82 KB PDF) [file pgen.0030235.sg001.pdf]

AATGTAATAATTTCTTTGTTTGAGTAGTTGGCTTAGCACTTAGTAGTAAGT
GAAGTTTTACATGTTAAAACAGAGTATGAGGTTTTAGCAAAGTAAATGA

-*HPRT* cassette-

ATGACATGTTACAGGGCTTCTCTAAATAGTGCCTGAGAATTTTTTTAAAAA
TCATTTTCTTTCTATAACAGGCATAAGAAATTCATCATACTCATACCAATC
TACACAAGAGTGAGATCATGGTACCACCTACATATTCATCTATAATAAAAC
CAAGAAAATAAGAAAATACTTTATATATAAATATTTGTTTTTATGAAGCCA
TAAAACCTAACTATATAATTTGGGGGAAAAA

Figure S1.

Sequences at the site replacing the *PWScr* with the *HPRT* cassette.

The primary sequence of the 5’- (chr7:67,076,064) and 3’- (chr7:66,883,161) regions flanking the inserted *HPRT* cassette (Genbank accession number EU233428). The sequence of *Ipw* exon H is underlined. The sequence positions correspond to the UCSC Genome Browser (mouse, July 2007 assembly). The compiled deleted region is estimated to span ~193 kb; however, because the gap size is only estimated (~50 kb chr7:66,952,205-67,002,206), the region deleted in our mouse model could span from 143 to ≥193 kb.
