# Supplementary material for: Deletion of the MBII-85 snoRNA Gene Cluster in Mice Results in Postnatal Growth Retardation
Source: PLoS Genet. 2007 Dec 28;3(12):e235. doi: 10.1371/journal.pgen.0030235 (PMC2323313; doi:10.1371/journal.pgen.0030235)
Supplement: Figure S2 — (A, B) Growth dynamics of mice in 126SV x C57BL/6 x FVB/N genetic crosses (∼50% FVB/N contribution) beginning at postnatal day 1. (A) Growth dynamics of 11 investigated males. The yellow line corresponds to the weight gain of 8 PWScrm+/p− males. The black line corresponds to the weight gain of 3 wild-type males. (B) Growth dynamics of 21 investigated female mice. The yellow line corresponds to the weight gain of 13 PWScrm+/p− females. The black line corresponds to the weight gain of 8 wild-type females. (C, D) Growth dynamics of mice in 126SV x C57BL/6 x BALB/c genetic crosses (∼50% BALB/c contribution) beginning at postnatal week 6. (C) Growth dynamics of 100 analyzed male mice. The yellow line corresponds to weight gain of 47 PWScrm+/p− males. The black line corresponds to 53 PWScrm+/p+ males. (D) Growth dynamics of 102 female mice. The yellow line corresponds to the weight gain of 51 PWScrm+/p− females. The black line corresponds to the weight gain of 51 PWScrm+/p+ females. In all cases, black error bars exhibit statistically significant intervals (confidence level 95%, p=0.05). (109 KB PDF) [file pgen.0030235.sg002.pdf]

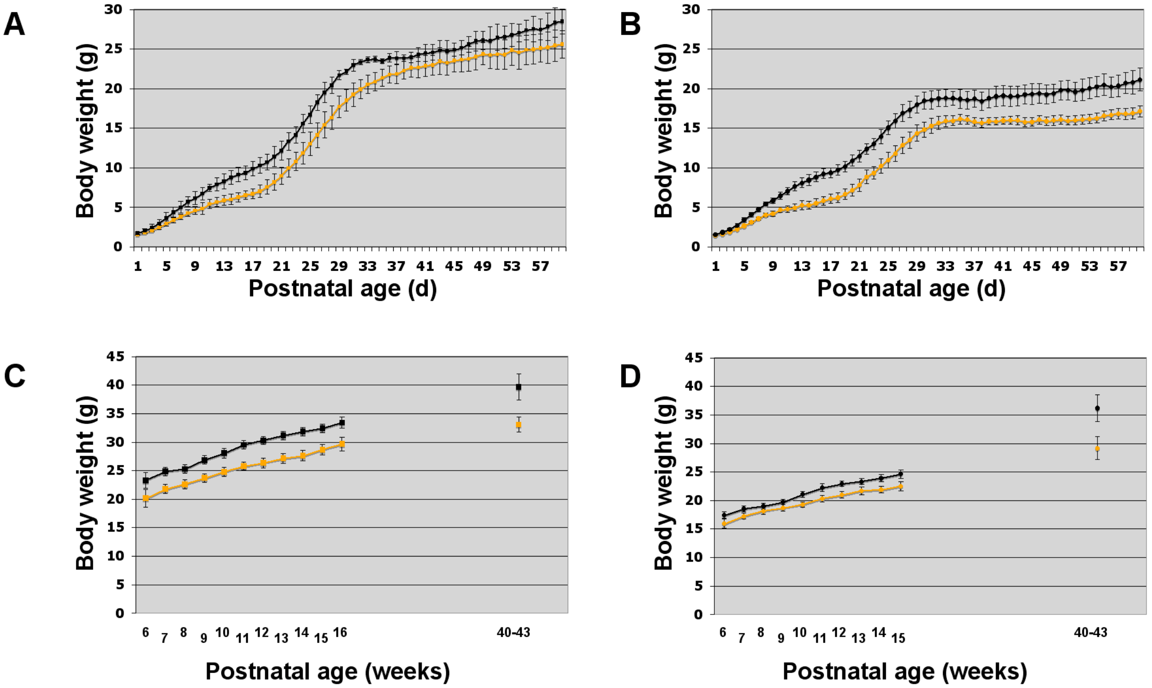

Figure S2.

Growth retardation in *PWScr*$^{m+/p-}$ mice in FVB/N and BALB/c genetic crosses.

A, B. Growth dynamics of mice in 126SV x C57BL/6 x FVB/N genetic crosses (~50% FVB/N contribution) beginning at postnatal day 1. **A.** Growth dynamics of 11 investigated males. The yellow line corresponds to the weight gain of 8 *PWScr*$^{m+/p-}$ males. The black line corresponds to the weight gain of 3 wild-type males. **B.** Growth dynamics of 21 investigated female mice. The yellow line corresponds to the weight gain of 13 *PWScr*$^{m+/p-}$ females. The black line corresponds to the weight gain of 8 wild-type females. **C, D.** Growth dynamics of mice in 126SV x C57BL/6 x BALB/c genetic crosses (~50% BALB/c contribution) beginning at postnatal week 6. **C.** Growth dynamics of 100 analyzed male mice. The yellow line corresponds to weight gain of 47 *PWScr*$^{m+/p-}$ males. The black line corresponds to 53 *PWScr*$^{m+/p+}$ males. **D.** Growth dynamics of 102 female mice. The yellow line corresponds to the weight gain of 51 *PWScr*$^{m+/p-}$ females. The black line corresponds to the weight gain of 51 *PWScr*$^{m+/p+}$ females. In all cases, black error bars exhibit statistically significant intervals (confidence level 95%, p=0.05).
