# Supplementary material for: Deletion of the MBII-85 snoRNA Gene Cluster in Mice Results in Postnatal Growth Retardation
Source: PLoS Genet. 2007 Dec 28;3(12):e235. doi: 10.1371/journal.pgen.0030235 (PMC2323313; doi:10.1371/journal.pgen.0030235)
Supplement: Table S1 — (27 KB DOC) [file pgen.0030235.st001.doc]

**Table S1.**

**Sequences of oligonucleotides used in this study**

*Oligonucleotides for targeting vectors construction:*

5’FLAdir: tttcctttgtcgacTTTCCCAACCCTTCTAAGTCGT,

5’FLArev: tttcctttatcgatCATTTACTTTGCTAAAACCTCATAC

5'FLBdir: tttcctttgtcgacGGCCACCACATTTGCTCTTTG

5'FLBrev: tttcctttgcggccgcTCTCTGAAGCAGCACTGCAGGG

3'FLBdirN: tttcctttagcgctATGACATGTTACAGGGCTTCTC

3'FLBrev: tttcctttgtcgacTAATCATCCATACAGCCAAACC

3'FLAdir: tttcctttgcggccgcTTCTCTTCCTTCTCCCCATCTTC

3'FLArev: tttcctttggcgcgccTCAGGCACTATTTAGAGAAGCCC

*Oligonucleotides for 5’HR probe cloning:*

5'PRAdir: tttcctttaagcttGAGACTGCTATTATATTAATCCC

5'PRArev: tttcctttgtcgacACCCAGATCCTAAAGGTTCTA

5'PRBdir: tttcctttgtcgaCTCCCAGATAGTTCCTACCCTT

5'PRBrev: tttcctttatcgaTAGGACCAAGTGACACATGAAT

*Oligonucleotides for 3’HR probe cloning:*

3'PRAdir: tttcctttaagcttAACAGTTTGATAATGCTAAGTGA

3'PRArev: tttcctttgtcgacTAGCTCTGGGATATACTTAGGT

3'PRBdir: tttcctttgtcgacTGAAGCATAGAATTAACTCGTA

3'PRBrev: tttcctttatcgaTATTGTTTGAACTCCTTAATGAC

Sequences in small case were included in primers to facilitate subcloning.

*Oligonucleotides for PCR screening of targeted ES colonies:*

85–5'screen2d: TCGAGGAACTTGTAAATCATAG

85–5'screen2r: TGTGTAGCGCCAAGTGCCC

85-5'screen3d: TTTTTCCTCTTCAGTTTTATTTC

85-5'screen3r: GAAAAGCGCCTCCCCTACC

85-3'screen1d: CTGGTCATGCATGCCTGGAAT

85-3'screen1r: GGGCCCTACTATCTGCTCCTTACA

85-3'screen2d: AACTTGCCCCTTGCTCCATACC

85-3'screen2r: TTTACCTGTTATTTCCTTTTGTGC

*Oligonucleotides for amplification and sequencing of the PWScr deleted allele:*

MB85seqD1: TAATAATTTCTTTGTTTGAGTAGT

MB85seqR1: GATTATTTTCCTCTGTGTCG

*Oligonucleotides for PWScr mice genotyping:*

MB85deld1: gAggCCATAAACAAgAAgACTAAA

MB85delr1: AgCTTgCgACCTTgACCATCT

*Oligonucleotides for RT-PCR analysis:*

Mkrnd2: gggCAgTgCAAggAgggCgATAAC

Mkrnr2: AggCATTgTCCCCgggCAgCATAg

Ndnd1: gCCCACCAgCCCCAgAgTCCA

Ndnr1: CCCCgCggCCCTTCACATAgAT

Magelr1: TggCACggTTgATgATgTCTAAgC

Mageld1: CTTggAgggCCCTAgCACTTCAC

Fratd1: gAgCgCCCCgAAATgCCACACTTg

Fratr1: TgCTgCCTCCCgCCCTACACTCAT

SnrpnD1: gACgCTTggTTCTgAggAgTgATT

SnrpnR1: CgATgCAgggCTATTAACAAACAA

Ube3Ad1: gTCCgCCCACCTAATCCTCTCgTC

Ube3Ar1: gCggCCgCCTCACTggTCCTT

Atp10d1: AACCACCTgggCTgCCTTgTCTTC

Atp10r1: TTggCgCCgCTTCAggTTggTCTC

GAPDHd1: ggggTgAggCCggTgCTgAgT

GAPDHr1: TTgggggCCgAgTTgggATAgg

βACTd1: TAAggCCAACCgTgAAAAgATgAC

βACTr1: ACCgCTCgTTgCCAATAgTgATg

*Oligonucleotides for cDNA probes used in Northern blot hybridization:*

NDNd1: gCCCACCAgCCCCAgAgTCCA

NDNr2: ACGTTTATTTATGGTGGGGTTGC

UBE3Ad1: gTCCgCCCACCTAATCCTCTCgTC

UBE3Ar2: TGGCATCATCATCATTCACTA

ATP10d1: AACCACCTgggCTgCCTTgTCTTC

ATP10r2: GGCCTGAGATCCCAACACCTAC

IPWFd1: GGGTGCCCTTACTTCCATCTA

IPWFr1: CTTTCCAAAATTGCTTCAGAGT

*Oligonucleotides used for snoRNAs detection in Northern blot hybridization:*

MBII-436: gCTCAATTTTTgAAATCATTATATTCAgACAAgg

MBII-13: CTTCAgAgTAATCATTTTgAgCATCATTTC

MBII-85: TTCCGATGAGAGTGGCGGTACAGA

MBII-52: CCTCAGCGTAATCCTATTGAGCATGAA

5.8S RNA: TCCTgCAATTCACATTAATTCTCgCAgCTAgC
