# Supplementary material for: Deletion of the MBII-85 snoRNA Gene Cluster in Mice Results in Postnatal Growth Retardation
Source: PLoS Genet. 2007 Dec 28;3(12):e235. doi: 10.1371/journal.pgen.0030235 (PMC2323313; doi:10.1371/journal.pgen.0030235)
Supplement: Table S2 — P1 – P8 indicate the corresponding postnatal days. N is the number of mice investigated from each genotype. Mean is the average mouse weight per category in grams. Statistically significant differences (p < 0.05) are indicated in bold. (76 KB DOC) [file pgen.0030235.st002.doc]

**Table S2.**

| Male | genotype | N= | Mean | Std. Dev. | Std. Err. | F-Value | P-Value |
| --- | --- | --- | --- | --- | --- | --- | --- |
| P1 | *PWScr^m+/p+^* | 21 | 1.43 | 0.132 | 0.029 | 0.006 | 0.9401 |
|  | *PWScr^m+/p-^* | 17 | 1.433 | 0.146 | 0.035 |  |  |
| P2 | *PWScr^m+/p+^* | 21 | 1.545 | 0.16 | 0.035 | 0.995 | 0.3251 |
|  | *PWScr^m+/p-^* | 17 | 1.496 | 0.133 | 0.032 |  |  |
| P3 | *PWScr^m+/p+^* | 21 | 1.765 | 0.22 | 0.048 | 3.077 | 0.0879 |
|  | *PWScr^m+/p-^* | 17 | 1.652 | 0.168 | 0.041 |  |  |
| P4 | *PWScr^m+/p+^* | 21 | 2.137 | 0.297 | 0.065 | 3.601 | 0.0658 |
|  | *PWScr^m+/p-^* | 17 | 1.972 | 0.222 | 0.054 |  |  |
| P5 | *PWScr^m+/p+^* | 21 | 2.578 | 0.344 | 0.075 | 5.675 | **0.0226** |
|  | *PWScr^m+/p-^* | 17 | 2.343 | 0.241 | 0.058 |  |  |
| P6 | *PWScr^m+/p+^* | 21 | 3.046 | 0.397 | 0.087 | 8.637 | **0.0057** |
|  | *PWScr^m+/p-^* | 17 | 2.708 | 0.289 | 0.07 |  |  |
| P7 | *PWScr^m+/p+^* | 21 | 3.551 | 0.436 | 0.095 | 14.332 | **0.0006** |
|  | *PWScr^m+/p-^* | 17 | 3.087 | 0.283 | 0.069 |  |  |
| P8 | *PWScr^m+/p+^* | 21 | 4.036 | 0.526 | 0.115 | 16.647 | **0.0002** |
|  | *PWScr^m+/p-^* | 17 | 3.442 | 0.319 | 0.077 |  |  |
| Female |  |  |  |  |  |  |  |
| P1 | *PWScr^m+/p+^* | 20 | 1.399 | 0.113 | 0.025 | 1.53 | 0.2231 |
|  | *PWScr^m+/p-^* | 23 | 1.352 | 0.132 | 0.028 |  |  |
| P2 | *PWScr^m+/p+^* | 20 | 1.503 | 0.146 | 0.033 | 2.51 | 0.1208 |
|  | *PWScr^m+/p-^* | 23 | 1.431 | 0.15 | 0.031 |  |  |
| P3 | *PWScr^m+/p+^* | 20 | 1.669 | 0.222 | 0.05 | 1.608 | 0.2119 |
|  | *PWScr^m+/p-^* | 23 | 1.587 | 0.202 | 0.042 |  |  |
| P4 | *PWScr^m+/p+^* | 20 | 2.004 | 0.315 | 0.07 | 2.814 | 0.1011 |
|  | *PWScr^m+/p-^* | 23 | 1.864 | 0.229 | 0.048 |  |  |
| P5 | *PWScr^m+/p+^* | 20 | 2.447 | 0.398 | 0.089 | 3.404 | 0.0723 |
|  | *PWScr^m+/p-^* | 23 | 2.24 | 0.34 | 0.071 |  |  |
| P6 | *PWScr^m+/p+^* | 20 | 2.913 | 0.478 | 0.107 | 8.042 | **0.0071** |
|  | *PWScr^m+/p-^* | 23 | 2.547 | 0.366 | 0.076 |  |  |
| P7 | *PWScr^m+/p+^* | 20 | 3.458 | 0.488 | 0.109 | 15.74 | **0.0003** |
|  | *PWScr^m+/p-^* | 23 | 2.919 | 0.402 | 0.084 |  |  |
| P8 | *PWScr^m+/p+^* | 20 | 3.897 | 0.612 | 0.137 | 17.981 | **0.0001** |
|  | *PWScr^m+/p-^* | 23 | 3.199 | 0.466 | 0.097 |  |  |

**Statistical analysis (ANOVA) of early postnatal weigh gain for *PWScr^m+/p+^* and *PWScr^m+/p-^* mice separated by gender.** P1 – P8 indicate the corresponding postnatal days. N is the number of mice investigated from each genotype. Mean is the average mouse weight per category in grams. Statistically significant differences (p < 0.05) are indicated in bold.
