# Supplementary material for: Deletion of the MBII-85 snoRNA Gene Cluster in Mice Results in Postnatal Growth Retardation
Source: PLoS Genet. 2007 Dec 28;3(12):e235. doi: 10.1371/journal.pgen.0030235 (PMC2323313; doi:10.1371/journal.pgen.0030235)
Supplement: Table S3 — Twenty-nine crosses between PWScrm+/p− males and wild type BALB/c females resulted in 230 embryos/pups. Among those 106 were identified as PWScrm+/p+, 121 as PWScrm+/p− and 3 were not genotyped. We have performed 12 crosses between PWScrm−/p+ males and wild type BALB/c females and obtained 100 embryos/pups. Fifty were identified as PWScrm+/p+, 48 as PWScrm+/p− and 2 were not genotyped. (63 KB DOC) [file pgen.0030235.st003.doc]

Table S3.

| Male  and genotype | Total pups/embryos (stage) | *PWScrm+/p-* | *PWScrm+/p+* | Genotype not determined |
| --- | --- | --- | --- | --- |
| Nr 9 *PWScrm+/p-* | 17* (P1) | 12 | 4 | 1 |
| Nr 18 *PWScrm+/p-* | 10 (P2) | 4 | 5 | 1 |
| Nr 18 *PWScrm+/p-* | 5 (P3) | 3 | 2 |  |
| Nr 18 *PWScrm+/p-* | 8 (P>5) | 2 | 6 |  |
| Nr 18 *PWScrm+/p-* | 9 (P>5) | 6 | 3 |  |
| Nr 18 *PWScrm+/p-* | 6 (P>5) | 3 | 3 |  |
| Nr 33 *PWScrm-/p+* | 9 (P>5) | 5 | 4 |  |
| Nr 33 *PWScrm-/p+* | 7 (P>5) | 2 | 5 |  |
| Nr 39 *PWScrm-/p+* | 8 (E16.5) | 3 | 5 |  |
| Nr 39 *PWScrm-/p+* | 9 (E14.5) | 3 | 6 |  |
| Nr 40 *PWScrm-/p+* | 9 (E16.5) | 8 | 1 |  |
| Nr 40 *PWScrm-/p+* | 6 (P3) | 2 | 4 |  |
| Nr 40 *PWScrm-/p+* | 6 (P>5) | 3 | 3 |  |
| Nr 41 *PWScrm-/p+* | 8 (E14.5) | 2 | 6 |  |
| Nr 41 *PWScrm-/p+* | 11 (E16.5) | 6 | 5 |  |
| Nr 41 *PWScrm-/p+* | 7 (P1) | 5 | 2 |  |
| Nr 50 *PWScrm+/p-* | 8 (P3) | 5 | 3 |  |
| Nr 52 *PWScrm+/p-* | 6 (P3) | 2 | 4 |  |
| Nr 52 *PWScrm+/p-* | 9 (P>5) | 4 | 5 |  |
| Nr 52 *PWScrm+/p-* | 11 (P>5) | 6 | 5 |  |
| Nr 55 *PWScrm+/p-* | 8 (P>5) | 4 | 4 |  |
| Nr 55 *PWScrm+/p-* | 7 (P>5) | 6 | 1 |  |
| Nr 55 *PWScrm+/p-* | 6 (P>5) | 2 | 4 |  |
| Nr 55 *PWScrm+/p-* | 5 (P>5) | 4 | 1 |  |
| Nr 73 *PWScrm-/p+* | 9 (E16.5) | 5 | 2 | 2 |
| Nr 73 *PWScrm-/p+* | 11 (P2) | 4 | 7 |  |
| Nr 94 *PWScrm+/p-* | 8 (P2) | 5 | 3 |  |
| Nr 94 *PWScrm+/p-* | 19* (P>5) | 8 | 11 |  |
| Nr 94 *PWScrm+/p-* | 5 (P>5) | 2 | 3 |  |
| Nr 94 *PWScrm+/p-* | 6 (P>5) | 2 | 4 |  |
| Nr 94 *PWScrm+/p-* | 6 (P>5) | 2 | 4 |  |
| Nr 94 *PWScrm+/p-* | 6 (P>5) | 5 | 1 |  |
| Nr 94 *PWScrm+/p-* | 6 (P>5) | 4 | 2 |  |
| Nr 95 *PWScrm+/p-* | 11 (E16.5) | 2 | 9 |  |
| Nr 100 *PWScrm+/p-* | 11 (E14.5) | 6 | 4 | 1 |
| Nr 100 *PWScrm+/p-* | 10 (E16.5) | 5 | 5 |  |
| Nr 100 *PWScrm+/p-* | 15* (P>5) | 10 | 5 |  |
| Nr 100 *PWScrm+/p-* | 12 (P>5) | 7 | 5 |  |

*number of mice born from 2 females.

**Number of embryos/pups from several *PWScrm+/p-* and *PWScrm-/p+* male mice**

Twenty-nine crosses between *PWScrm+/p-* males and wild type BALB/c females resulted in 230 embryos/pups. Among those 106 were identified as *PWScrm+/p+*, 121 as *PWScrm+/p-* and 3 were not genotyped. We have performed 12 crosses between *PWScrm­/p+* males and wild type BALB/c females and obtained 100 embryos/pups. Fifty were identified as *PWScrm+/p+*, 48 as *PWScrm+/p-* and 2 were not genotyped.
